# Intensity-modulated radiation therapy (IMRT) vs. 3D conformal radiotherapy (3DCRT) in locally advanced rectal cancer (LARC): dosimetric comparison and clinical implications

**DOI:** 10.1186/1748-717X-5-17

**Published:** 2010-02-26

**Authors:** Leire Arbea, Luis Isaac Ramos, Rafael Martínez-Monge, Marta Moreno, Javier Aristu

**Affiliations:** 1 Department of Oncology, Clínica Universidad de Navarra, Pamplona, Spain

## Abstract

**Purpose:**

To compare target dose distribution, comformality, normal tissue avoidance, and irradiated body volume (IBV) in 3DCRT using classic anatomical landmarks (c3DCRT), 3DCRT fitting the PTV (f3DCRT), and intensity-modulated radiation therapy (IMRT) in patients with locally advanced rectal cancer (LARC).

**Materials and methods:**

Fifteen patients with LARC underwent c3DCRT, f3DCRT, and IMRT planning. Target definition followed the recommendations of the ICRU reports No. 50 and 62. OAR (SB and bladder) constraints were D5 ≤ 50 Gy and Dmax < 55 Gy. PTV dose prescription was defined as PTV95 ≥ 45 Gy and PTVmin ≥ 35 Gy. Target coverage was evaluated with the D95, Dmin, and Dmax. Target dose distribution and comformality was evaluated with the homogeneity indices (HI) and Conformity Index (CI). Normal tissue avoidance of OAR was evaluated with the D5 and V40. IBV at 5 Gy (V5), 10 Gy (V10), and 20 Gy (V20) were calculated.

**Results:**

The mean GTV95, CTV95, and PTV95 doses were significantly lower for IMRT plans. Target dose distribution was more inhomogeneous after IMRT planning and 3DCRTplans had significantly lower CI. The V40 and D5 values for OAR were significantly reduced in the IMRT plans .V5 was greater for IMRT than for f3DCRT planning (p < 0.05) and V20 was smaller for IMRT plans(p < 0.05).

**Conclusions:**

IMRT planning improves target conformity and decreases irradiation of the OAR at the expense of increased target heterogeneity. IMRT planning increases the IBV at 5 Gy or less but decreases the IBV at 20 Gy or more.

## Introduction

Preoperative chemoradiation (CRT) is the standard neoadjuvant treatment in patients with LARC (T3 and/or N+)[1]. The German CAO/ARO/AIO 94 trial confirmed that, compared to postoperative CRT in LARC, preoperative CRT produces significantly lower local recurrence rates, less acute and chronic toxicity, and an increased rate of sphincter preservation [2]. However, despite its improved compliance rate, preoperative CRT still results in considerable acute gastrointestinal toxicity. Acute grade 3 or greater diarrhea is observed in 12-25% of the patients, depending on the mode of 5-FU delivery. Combining new chemotherapy agents concurrently with preoperative radiation such as capecitabine and oxaliplatin has resulted in similar rates of acute toxicity [1,3-5]. Small bowel (SB) toxicity is increased with wider irradiated fields, higher radiation dosages, inappropriate irradiation techniques, and larger irradiated SB volumes[6]. The relationship between SB irradiation and grade 3 diarrhea has been observed at all dose levels during preoperative CRT[7], and some dosimetric quantifiers, such as V15 (the absolute volume of SB receiving at least 15 Gy) [8] have been postulated to represent a reliable cut-off during dose plan evaluation.

IMRT produces highly conformal dose distributions in the target volumes and minimizes the dose received by adjacent dose-limiting structures. This ability of IMRT to decrease bowel irradiation has been widely reported in gynecologic and prostate cancer studies([9,10]). However, dosimetric studies comparing IMRT and 3DCRT in LARC are scarce ([7,11,12]) and include small patient samples that range from 5 to 8.

We designed this planning study to compare the potential dosimetric advantages of IMRT and conventional 3DCRT using classic anatomical landmarks (c3DCRT) and 3DCRT fitting the field edges of the PTV (f3DCRT) in a larger patient sample of 15 individuals. Forty-five dosimetric plans were generated for analysis. The following planning results were evaluated and compared: target coverage and target dose distribution; comformality; normal tissue avoidance, and irradiated body volume.

## Materials and methods

Fifteen consecutive patients with LARC underwent c3DCRT, f3DCRT, and IMRT planning at the Radiation Oncology Division of the University of Navarre, Spain, from March 2003 to September 2003. Two patients had tumors arising in the upper third of the rectum, eight in the middle third, and five in the lower third. Ten tumors were staged by echoendoscopy as uT3N+ and five as uT3N0. Patients were immobilized in the prone position using a combination of a foam cushion and a prone head cushion. Setup marks were drawn on the patient's skin and the cushion after laser alignment. A non contrast-enhanced planning CT scan was performed using a diagnostic CT scanner (Somatron Plus 4, Siemens Oncology Care Systems, Heidelberg, Germany) with a flat table insert. Patients were instructed to have an empty bladder before CT scan. The scan extended from the L2 vertebral body to 2 cm below the perineum, and axial images were obtained at 5 mm intervals and imported to the planning system (Helax-TMS, Nucletron Scandinavia, Uppsala, Sweden).

Target definition followed the recommendations of the ICRU reports No. 50 and 62[13]. The GTV-T and GTV-N were delineated using information from the diagnostic CT and the Endoscopic Ultrasound (EUS). The clinical target volume (CTV) included the GTV-T and GTV-N (if any), the presacral nodes, the complet mesorectum and the common and internal iliac lymph nodes. The PTV was generated with an asymmetrical margin around the CTV. In areas in which the tumor was close to the SB and bladder, a 5-mm expansion was used while a 10-mm margin was used in the rest of the volume. The organ at risk volumes (OARVs) outlined were the bladder, the rectum from the sphincter to the sigmoid (including the GTV-T), and the SB. The SB was outlined 1 cm above and below the PTV, and the bladder was fully contoured.

For *c3DCRT *planning, a four-field technique using the classic anatomical references was used [14] regardeless of the PTV designed. The superior edge of AP-PA portals was placed between the sacral promontory and the L4-L5 interspace, and the inferior border was placed on the ischial tuberosities. If the tumor was located in the lower third of the rectum, the inferior edge was displaced inferiorly to include the perineum. The lateral borders of AP-PA portals were placed to provide adequate coverage of the pelvic sidewalls with a 1-cm margin. The posterior margin for lateral fields was placed 1.5-2.0 cm posterior to the anterior border of the sacrum. The anterior border of the lateral fields usually covered at least the posterior border of the vagina or the prostate, the anterior extent of the primary rectal tumor, and the anterior edge of the sacral promontory. Customized shielding was performed using 1-cm leaves.

In *f3DCRT *planning, the beams of a typical four-field arrangement were shaped to the PTV with 1-cm leaves.

A 45 Gy dose delivered with 15 MV photons was prescribed to the PTV_95 _(the minimal dose received by 95% of the PTV) for the 3DCRT plans. Photon dose calculation in tridimensional radiotherapy planning was made using the pencil beam with the Iwasaki algorithm to correct inhomogeneities [15]*. IMRT *planning procedure has been described previously [16,17]. Treatment planning was performed using the KonRad inverse planning system, version 2. 0 (Siemens Oncology Care Systems). Seven coplanar equi-spaced fields (gantry angles 0°, 51°, 103°, 154°, 206°, 257°, and 308°) were generated with a median of 51 segments (range, 44 to 67). The isocenter was placed at the geometric center of the PTV. The hierarchy of dose constraints and dose prescription was as follows: first, SB; second, PTV; third, bladder. Plans were accepted when the PTV_95 _was ≥ 45 Gy, the dose received by 5% of the SB (SB D_5_) was ≤ 50 Gy, the PTV_min _(minimal dose to the PTV) was ≥ 35 Gy, and maximal SB dose (SB_max_) was 55 Gy. Dose constraints for the bladder included a maximal dose (Bladder_max_) of 55 Gy and a minimal dose received by 5% of the bladder (Bladder D_5_) of 50 Gy. No specific rectum or external volume constraints were used. IMRT was delivered with 15 MV photons generated in a Mevatron Primus and Oncor linear accelerator (Siemens Oncology Care Systems, Concord, CA). The dose calculation algorithm was also pencil beam with 0.25 cm of voxel size. Konrad calculate the optimum fluence based on physical constraints followed by aperture calculation of the segments [18].

### Dosimetric Evaluation

The target coverage and target dose distribution were evaluated in the GTV, CTV, and PTV obtaining the following parameters for each of the three treatment modalities: minimal target dose (GTV_min_, CTV_min_, PTV_min_), maximal target dose (GTV_max_, CTV_max_, PTV_max_) calculated in all voxels of target volume, minimal dose to 95% of the volume (GTV_95_, CTV_95_, PTV_95_), and homogeneity index (HIG_TV_, HIC_TV_, HIP_TV_). The homogeneity index (HI) was defined as the standard deviation of the normalized differential DVH curve [19] within a target volume.

The degree of comformality was evaluated with a conformity index (CI) that was defined as the ratio between the target volume (PTV) and the irradiated volume at specified prescription dose (Vol PTV/Vol IR95%) [20].

Normal tissue (bladder, SB, and rectum) avoidance was evaluated using the following parameters: minimal dose received by 5% or less of the volume (Bladder D_5_, SB D_5_, Rectum D_5_) and absolute organ volume receiving 40 Gy or more (Bladder V_40_, SB V_40_, Rectum V_40_).

Finally, irradiated body volumes at the dose levels of 5 Gy (V_5_), 10 Gy (V_10_), and 20 Gy (V_20_) were calculated for each treatment modality. We also calculated the average cut-off point doses at which the irradiated body volumes were significantly different between treatment modalities.

Plans were compared using the Kruskal Wallis test. If positive results, a paired U-Mann-Whitney test was applied with the statement that the IMRT is a reference. We compare IMRT vs f3DCRT and IMRT vs c3DCRT and the differences were considered as statistically significant at the p ≤ 0.05 level.

## Results

### Target Coverage and Target Dose Distribution

**Figure 1:**
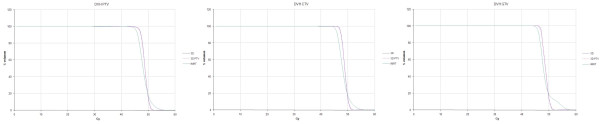
**Target dose volume histograms comparison**. DVH: dose-volume histogram. GTV: gross tumor volume. CTV: clinical target volume. PTV: planning target volume. c3DCRT: conventional tridimensional conformal radiotherapy (blue line). f3DCRT: modificated tridimensional conformal radiotherapy (pink line). IMRT: intensity modulated radiation therapy (green line).

The c3DCRT and f3DCRT plans met the prescription goal of PTV_95 _≥ 45 Gy in all cases. However, the prescription goal of PTV_95 _≥ 45 Gy was not reached in two IMRT patients (44.8 Gy and 44.4 Gy, respectively). CTV_95 _was lower than 45 Gy in one IMRT case (44.9 Gy), and GTV_95 _was ≥ 45 Gy in all cases. The mean GTV_95_, CTV_95_, and PTV_95 _doses were found to be significantly lower for IMRT plans than for c3DCRT and f3DCRT plans. Table 1 list the D_95_, D_min_, and D_max _values for the target volumes. DVHs of the target volumes with the three different techniques are shown in Figure 1. Finally, the dose distribution across the target volumes was less homogeneous after IMRT planning than after c3DCRT or f3DCRT planning. This difference was statistically significant (p < 0.05) for all the volumes analyzed (Table 2).

**Table 1:** Dosimetric summary of target volumes

		D_95 _(Gy)			Dmin (Gy)			Dmax(Gy)		
		c3DCRT	f3DCRT	IMRT	c3DCRT	f3DCRT	IMRT	c3DCRT	f3DCRT	IMRT
GTV	mean	47.5	47.5	46.9	46.7	46.6	45.1	50.2	50.3	51.6
	SD	0.6	0.7	2.3	0.4	0.6	2.0	1.0	1.0	2.7
	*p*	*p_1 _< 0.05*	*p_2 _< 0.05*	Ref	*p_1 _< 0.05*	*p_2 _< 0.05*	*Ref*	*p_1 _=0.2*	*p_2 _=0.3*	Ref
PTV	mean	47.1	46.9	45.7	39.5	42.2	39.2	51.1	51.2	54.0
	SD	0.5	0.5	0.8	4.2	1.2	1.7	0.6	0.5	2.6
	*p*	*p_1 _< 0.05*	*p_2 _< 0.05*	Ref	*p1 *= 0.4	*p_2 _< 0.05*	*Ref*	*p_1 _< 0.05*	*p_2 _< 0.05*	Ref

c3DCRT: classic tridimensional conformal radiotherapy;

f3DCRT: fitting tridimensional conformal radiotherapy,

IMRT: intensity modulated radiation therapy;

D_95_: minimal dose to 95% of the volume;

GTV: gross tumor volume;

PTV: planning target volume;

SD: Standard deviation;

p1: c3DCRT vs IMRT;

p2: f3DCRT vs IMRT;

Ref:Referencevalue

**Table 2:** Homogeneity and Conformity Index of Target Volumes

		HI			CI		
		c3DCRT	f3DCRT	IMRT	c3DCRT	f3DCRT	IMRT
GTV	mean	0.8	0.8	1.1			
	SD	0.2	0.2	0.3			
	*p*	*p_1 _< 0.05*	*p_2 _< 0.05*	Ref			
PTV	mean	1.1	1.1	1.6	0.5	0.6	0.8
	SD	0.1	0.2	0.3	0.1	0.1	0.1
	*p*	*p_1 _< 0.05*	*p_2 _< 0.05*	Ref	*p_1 _< 0.05*	*p_2 _< 0.05*	Ref

HI: homogeneity index;

CI: Conformity index

c3DCRT: classic tridimensional conformal radiotherapy;

f3DCRT: fitting tridimensional conformal radiotherapy,

IMRT: intensity modulated radiation therapy;

GTV: gross tumor volume;

CTV: clinical target volume;

PTV: planning target volume;

*p_1: _*c3DCRT vs IMRT_;_

*p_2: _*f3DCRT vs IMRT;

Ref:Referencevalue

### Comformality

**Figure 2:**
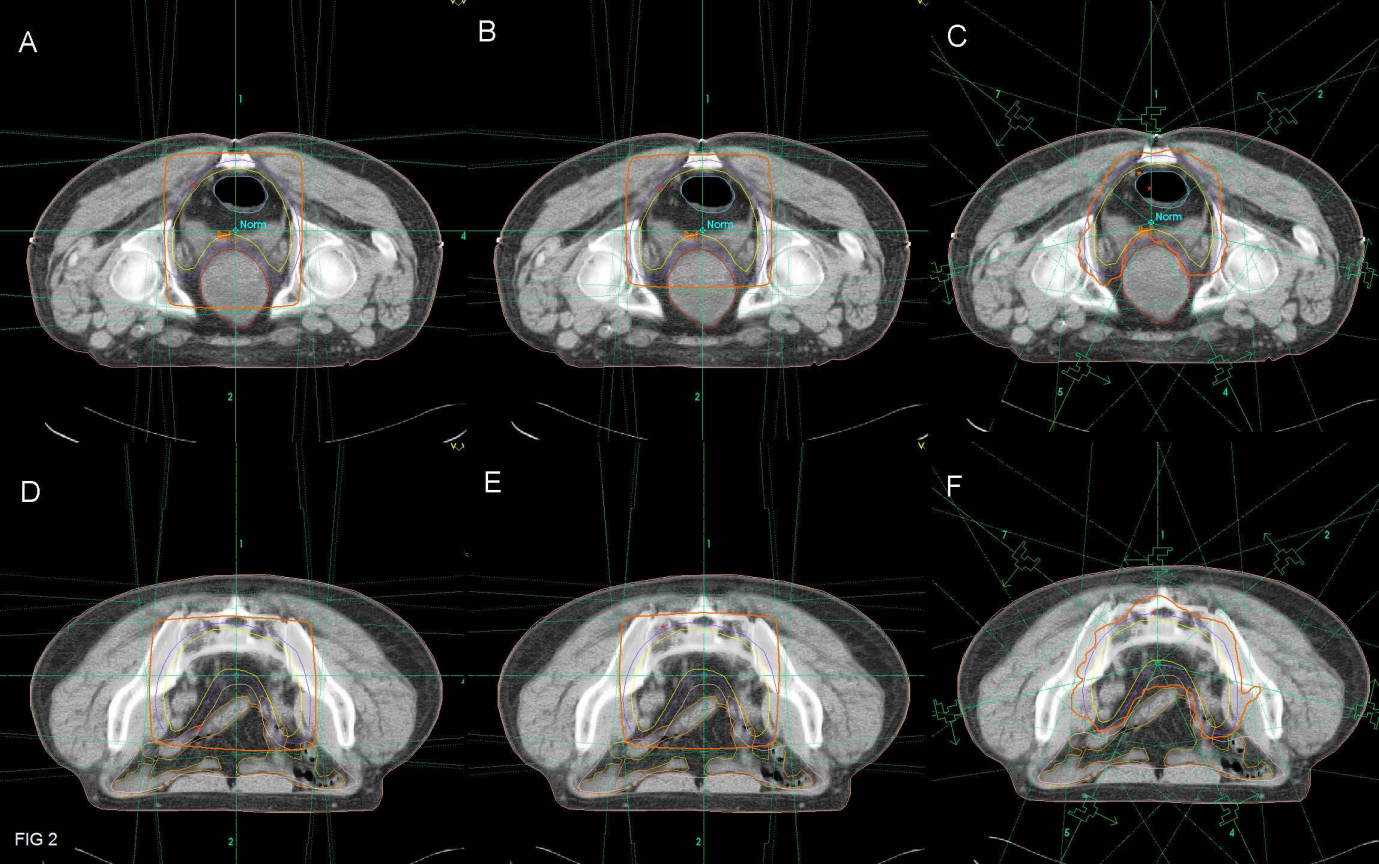
**Isodose distribution in a patient with a uT3N+ medial rectal cancer**. planned with c3DCRT (A and D), f3DCRT (B and E) and IMRT (C and F). The. orange line represents the 95% isodose. c3DCRT: conventional tridimensional conformal radiotherapy. f3DCRT: modificated tridimensional conformal radiotherapy. IMRT: intensity modulated radiation therapy.

The median volume of the PTV contoured in the 15 patients was 1211.6 cc (range, 870.2 to 1694.7 cc). IMRT planning had the highest level of comformality compared to the 3DCRT plans (Table 2). The average CI of the IMRT plans was 0.8, and the average CI of c3DCRT and f3DCRT planning were 0.5 and 0.6, respectively, (p < 0.05). Figure 2 shows representative axial CT slides that show the isodose distributions obtained with the three treatment modalities. Better dose conformation of the target volumes was observed after IMRT planning.

### Normal Tissue Avoidance

**Figure 3:**
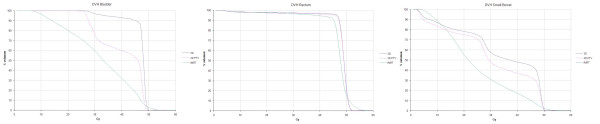
**Organ at risk dose volume histograms comparisson**. c3DCRT: conventional tridimensional conformal radiotherapy (blue line). f3DCRT: modificated tridimensional conformal radiotherapy (pink line). IMRT: intensity modulated radiation therapy (green line).

Table 3 and Figure 3 summarize the mean D5 and V40 values for the bladder, SB, and rectum. IMRT planning produced significantly lower D5 and V40 values for the bladder and the SB (p < 0.05). However, rectal values were not statistically different.

**Table 3:** OARV Parameters

		D_5 _(Gy)			V_40 _(cc)		
		c3DCRT	f3DCRT	IMRT	c3DCRT	f3DCRT	IMRT
BLADDER	mean	48.8	48.4	46.2	94.7	60.9	34.4
	SD	0.5	0.5	4.1	66.2	26.5	24.9
	*p*	*p_1 _< 0.05*	*p_2 _= 0.06*	Ref	*p_1 _< 0.05*	*p_2 _< 0.05*	Ref
SMALL BOWEL	mean	49.2	49.0	46.2	178.3	140.3	68.9
	SD	0.5	0.8	4.1	136.6	120.7	63.5
	*p*	*p_1 _< 0.05*	*p_2 _< 0.05*	Ref	*p_1 _< 0.05*	*p_2 _< 0.05*	Ref
RECTUM	mean	50.7	50.8	50.6	162.3		157.7
	SD	0.6	0.6	2.3	55.7	56.5	54.8
	*p*	*p_1 _= 0.8*	*p_2 _= 0.7*	Ref	*p_1 _= 0.8*	*p_2 _= 0.9*	Ref

OARV: Organs at risk volume

c3DCRT: classic tridimensional conformal radiotherapy;

f3DCRT: fitting tridimensional conformal radiotherapy,

IMRT: intensity modulated radiation therapy;

D5: minimal dose received by 5% of the OARV;

V40: volume receiving ≥ 40 Gy

GTV: gross tumor volume;

PTV: planning target volume;

*p_1: _*c3DCRT vs IMRT_;_

*p_2: _*f3DCRT vs IMRT;

Ref: Reference value

### Irradiated Body Volume

**Figure 4:**
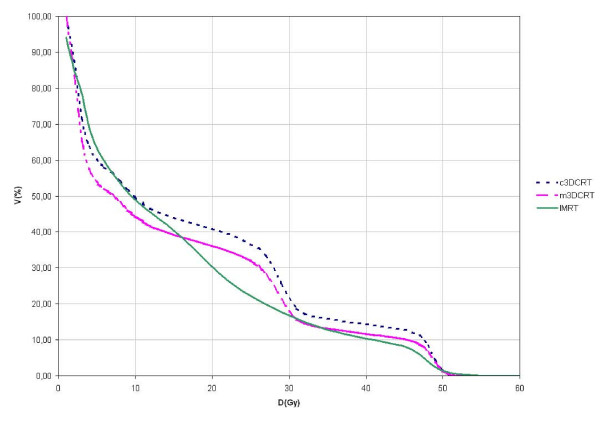
**Irradiated body volume dose-volume histograms**. c3DCRT: conventional tridimensional conformal radiotherapy (blue line). f3DCRT: modificated tridimensional conformal radiotherapy (pink line). IMRT: intensity modulated radiation therapy (green line).

The body volume receiving ≥ 5 Gy (V5) was significantly larger after IMRT planning than after f3DCRT planning (p < 0.05), but no statistical differences were found between IMRT and c3DCRT planning. No differences in V10 were observed among the 3 treatment modalities. However, V20 was significantly smaller after IMRT planning (Table 4). Volumetric analysis revealed that when isodoses are less than 8.4 Gy(95 CI: 6.2-10.6), the volumes of the isodoses from IMRT plans are larger than the isodoses volumes from c3DCRT plans and when isodoses are more than 15 Gy(95 CI:13.8-16.4), the isodose volumes from IMRT plans are smaller than the f3DCRT isodoses volumes (Figure 4).

**Table 4:** Irradiated Body Volume

	c3DCRT	f3DCRT	IMRT
V5 (cc)	11484	10302	12164
	*p_1 _= 0.43*	*p_2 _< 0.05*	Ref
V10 (cc)	9419	8416	9232
	*p_1 _= 0.9*	*p_2 _= 0.1*	Ref
V20 (cc)	7781	6881	5764
	*p_1 _< 0.05*	*p_2 _< 0.05*	Ref

c3DCRT: classic tridimensional conformal radiotherapy;

f3DCRT: fitting tridimensional conformal radiotherapy,

IMRT: intensity modulated radiation therapy;

V5: body volume receiving ≥ 5 Gy;

V10: body volume receiving ≥ 10 Gy;

V20: body volume receiving ≥ 20 Gy;

*p_1: _*c3DCRT vs IMRT_;_

*p_2: _*f3DCRT vs IMRT;

Ref: Reference value

## Discussion

This study was designed to compare the degree of target coverage and target dose distribution, comformality, normal tissue avoidance, and amount of irradiated body volume in IMRT, c3DCRT, and f3DCRT.

### Target Coverage and Dose Distribution

The IMRT plans failed to meet the prescription goal of PTV_95 _≥ 45 Gy in two out of 15 cases (44.8 Gy and 44.4 Gy), although the deviation was minimal (-0.4% and -1.3%, respectively). These minor deviations were the result of the normal tissue dose constraints. Duthoy et al. [11] compared intensity-modulated arc therapy (IMAT) with 3DCRT in LARC and did not observe differences in the coverage of the PTV.

The limitations and potential difficulties inherent to IMRT in the treatment of rectal cancer, i.e., organ motion, volume variability, dose inhomogeneity, and integral dose, must be considered. The rapid dose drop-off beyond target volumes, characteristic of IMRT, the internal target and organ at risk motion, and volume variability make treatment success higher dependent on accurate determination of target position, shape, and size, than in 3DCRT.

Rectal organ motion has been described almost solely in patients treated for prostate and bladder cancer. In these studies, rectal volume changes were observed during treatment, especially in the anterior wall and upper half of the rectum[21-25]. Nuyttens et al.[26] studied the variability of the CTV in rectal cancer due to internal organ motion during adjuvant treatment, but no data have been published on the variability of the rectal wall affected by tumor. The variation of rectal wall in patients with LARC would be probably smaller due to the fixation that can be confirmed by digital rectal examination in 88% of stage II and III tumors [27]. The influence of SB motion in IMRT for rectal cancer has been studied by Nuyttens et al [28]. In the preoperative setting, the SB is located in the superior pelvis where the posterior, lateral and anterior borders of the CTV are all very stable, therefore, the CTV is not probably influenced by SB motion and volume variability.

It is important to reably know the magnitude of internal organ motion, in order to assume a minimal variability to assure clinical reproducibility.

Based on these data, IMRT treatment planing goal have to be the coverage of prescribed dose in the 95% of PTV, and image verification becomes crucial.

Homogeneity is another issue that have to be explored in IMRT plans. In our study, target dose distribution across the GTV, CTV, and PTV was less homogeneous after IMRT planning than after c3DCRT or f3DCRT. The standard deviation of the normalized differential DVH curve across the PTV was 1.59 for IMRT, 1.12 for f3DCRT, and 1.10 for c3DCRT (p < 0.05). These results have been previously observed by other authors[12]. Other reports, however, have not shown an increased heterogeneity across the target volume with IMRT plans [7]. This fact may be relationated with the objective of IMRT; if the goal is uniformity, IMRT achieve more homogeneus plans, but if the goal is coverage, it could be at the expense of more inhomogeneity. In these cases, IMRT planning results in a trade-off between the coverage of the target, avoidance of adjacent healthy structures, and the inhomogeneity of the dose within the target. The consequences of non-uniform dose distributions in small target sub-volumes may not be deleterious. Tumor control has been related to the mean dose rather than to the minimum target absorbed dose when the dose uniformity is low, and more sub-volumes within the target volume may be advantageous [29]. Moreover, when inhomogeneity is present, it is important discriminate between overdosage and underdosage. If underdosing is observed, it is important to know the magnitude, location, and volume of the low dose regions. A modest number of colds spots (small volumes with moderate low dose) may not reduce tumor response or tumor control probability [30,31]. Our plans were visually checked to determine the location of cold and hot spots. The regions receiving 45 Gy or less were very small and were predominantly located on the anterior portion of the external iliac nodal region, an area with a low probability of tumor involvement. We also checked hot spots within the PTV to make sure the location out of some normal structures (i.e. sacral plexus) that are relationated with toxicity.

### SB Avoidance

IMRT plans produced a vast reduction in the mean SB V40. The volume of SB receiving ≥ 40 Gy with IMRT was roughly one third of the SB V40 irradiated with c3DCRT (68.9 cc vs. 178.3 cc, p < 0.05) and one-half of the SB irradiated with f3DCRT (68.9 cc vs. 140.3 cc, p < 0.05). The same findings were obtained when the fractional SB D5 was used instead. The c3DCRT and f3DCRT plans had higher SB D5 values than the IMRT plan (49.2 Gy vs. 46.2 Gy, p < 0.05; 49.0 Gy vs. 46.2 Gy, p < 0.05; respectively). When Duthoy et al. [11] compared intensity-modulated arc therapy (IMAT) with 3DCRT in LARC, the mean dose to the SB was significantly lower for the IMAT. A small retrospective planning study recently published by Guerrero et al. [12] compared the dosimetric distributions generated by IMRT and conventional 3DCRT plans in 5 patients. The results showed that the bowel volume irradiated to 45 Gy and 50 Gy was significantly reduced with IMRT. Tho et al. [7] performed additional IMRT planning in 8 LARC patients in whom the volume of SB included in the prescription isodose generated with 3DCRT was too large. Inverse planning reduced the median dose to the SB by 5.1 Gy (p = 0.008), as well as the individual volumes of SB receiving high and low dose irradiation.

### Bladder Avoidance

IMRT also demonstrated a clear advantage in terms of bladder sparing. The volume of bladder receiving ≥ 40 Gy with IMRT was approximately one third of the bladder V40 irradiated with c3DCRT (34.4 cc vs. 94.7 cc, p < 0.05) and one half of the bladder irradiated with f3DCRT (34.4 cc vs. 60.9 cc, p < 0.05). The fractional bladder D5 for the c3DCRT and f3DCRT plans were higher than for IMRT planning (48.8 Gy vs. 46.2 Gy, p < 0.05; 48.4 Gy vs. 46.2 Gy, p < 0.05; respectively).

### Rectal Avoidance

No differences were observed in the rectum parameters V40 and D5. This last finding might be explained in part by the large volume of normal rectum included in the CTV due to the relatively large tumor size in our patient sample (median size = 9.7 cm). It would be interesting, for future studies, to discriminate between different thirds of the rectum, and explore differences in the dose reaching the distal third in cases of proximal rectal tumors; the dose in distal rectum could be lower and this could mean less likelihood of acute and cronic toxicity.

### Irradiated Volume

IMRT delivers a higher integral dose to the body because of leakage radiation resulting from the use of a greater number of fields and a increased number of monitor units. A larger volume of normal tissue is exposed to lower doses than with 3DCRT techniques [32-34] and this radiobiological peculiarity has the effect of increasing the risk of a second malignancy [35,36]. IMRT may increase the 10-year incidence of second malignancies from 1% in patients treated with 3DCRT to 1.75% in patients treated with IMRT [37]. Dorr et al.[38] investigated the radiation-related parameters influencing the development of second malignancies in 31,000 patients treated with radiotherapy. The majority of second tumors were observed within the margin region of the PTV (volume from 2.5 cm inside to 5 cm outside of the margin of the PTV) and in the volume receiving less than 6 Gy [36]. Although this issue is constantly debated and explored since the begining of IMRT, there is no data available in the context of LARC. Taking into account the benefits of IMRT and published data of the incidence of second malignancies and its relation with dose and irradiated volume, we feel comfortable with IMRT in rectal cancer patients. Additionally, in our study, the body volume receiving 5 Gy or more (V5) was significantly larger after IMRT than after f3DCRT (p < 0. 05), although no differences were observed for V10. Statistical analysis identified the 8.4 Gy dose point as the threshold at which the detrimental effect of IMRT on irradiated volume disappears.

In summary, our results suggest that a 7 field-IMRT technique may potentially enhance the therapeutic ratio by reducing SB and bladder toxicity. This potential to reduce the toxicity profile might allow the use of a larger fraction size, which might shorten the overall treatment duration and improve cost-effectiveness. We have recently reported a phase I-II trial of concurrent capecitabine and oxaliplatin with preoperative IMRT in patients with LARC. The maximal tolerated dose in this regimen was 47.5 Gy in 19 daily treatments with promising rates of favourable pathologic response [16].

## Competing interests

The authors declare that they have no competing interests.

## Authors' contributions

JA and MM: idea and concept. LA and LIR: design and development of study LIR: statistical analysis. LA: writing of manuscript and study coordinator. RMM and JA: final revision of manuscript. All authors read and approved the final manuscript
